# Hypertension testing and treatment in Uganda and Kenya through the SEARCH study: An implementation fidelity and outcome evaluation

**DOI:** 10.1371/journal.pone.0222801

**Published:** 2020-01-15

**Authors:** David J. Heller, Laura B. Balzer, Dhruv Kazi, Edwin D. Charlebois, Dalsone Kwarisiima, Florence Mwangwa, Vivek Jain, Prashant Kotwani, Gabriel Chamie, Craig R. Cohen, Tamara D. Clark, James Ayieko, Dathan M. Byonanabye, Maya Petersen, Moses R. Kamya, Diane Havlir, James G. Kahn

**Affiliations:** 1 Arnhold Institute for Global Health, New York, New York, United States of America; 2 University of Massachusetts, Amherst, Massachusetts, United States of America; 3 Beth Israel Deaconess Medical Center, Boston, Massachusetts, United States of America; 4 University of California, San Francisco, San Francisco, California, United States of America; 5 Infectious Diseases Research Collaboration, Kampala, Uganda; 6 Kenya Medical Research Institute (KEMRI), Nairobi, Kenya; 7 School of Public Health, Makerere University College of Health Sciences, Kampala, Uganda; 8 University of California Berkeley School of Public Health, Berkeley, California, United States of America; 9 School of Medicine, Makerere University College of Health Sciences, Kampala, Uganda; University of Ghana College of Health Sciences, GHANA

## Abstract

**Background:**

Hypertension (HTN) is the single leading risk factor for human mortality worldwide, and more prevalent in sub-Saharan Africa than any other region [1]–although resources for HTN screening, treatment, and control are few. Most regional pilot studies to leverage HIV programs for HTN control have achieved blood pressure control in half of participants or fewer [2,3,4]. But this control gap may be due to inconsistent delivery of services, rather than ineffective underlying interventions.

**Methods:**

We sought to evaluate the consistency of HTN program delivery within the SEARCH study (NCT01864603) among 95,000 adults in 32 rural communities in Uganda and Kenya from 2013–2016. To achieve this objective, we designed and performed a fidelity evaluation of the step-by-step process (cascade) of HTN care within SEARCH, calculating rates of HTN screening, linkage to care, and follow-up care. We evaluated SEARCH’s assessment of each participant’s HTN status against measured blood pressure and HTN history.

**Findings:**

SEARCH completed blood pressure screens on 91% of participants. SEARCH HTN screening was 91% sensitive and over 99% specific for HTN relative to measured blood pressure and patient history. 92% of participants screened HTN+ received clinic appointments, and 42% of persons with HTN linked to subsequent care. At follow-up, 82% of SEARCH clinic participants received blood pressure checks; 75% received medication appropriate for their blood pressure; 66% remained in care; and 46% had normal blood pressure at their most recent visit.

**Conclusion:**

The SEARCH study’s consistency in delivering screening and treatment services for HTN was generally high, but SEARCH could improve effectiveness in linking patients to care and achieving HTN control. Its model for implementing population-scale HTN testing and care through an existing HIV test-and-treat program–and protocol for evaluating the intervention’s stepwise fidelity and care outcomes–may be adapted, strengthened, and scaled up for use across multiple resource-limited settings.

## Introduction

Cardiovascular disease (CVD) is the leading cause of death worldwide, and hypertension (HTN) is the leading risk factor for both cardiovascular disease and all-cause mortality [1]. Between 2002–2012, deaths due to CVD grew more significantly than for any other condition in Sub-Saharan Africa [5]. Relative to the global age-standardized population [6], HTN afflicts some 30% of adults across sub-Saharan Africa, the highest prevalence worldwide [7], and is projected to affect up to 150 million persons by 2025 [8]. In Uganda, standardized adult HTN prevalence estimates range from 27% to 32% or greater [9,10,11], with disease awareness at 8% [10]. Data in Kenya are similar, with age-standardized prevalence of 25–26% [12,13] disease awareness of 16% [12], and control under 3% [12].

Previous work demonstrates community-level programs to screen and treat CVD risk factors in sub-Saharan Africa are efficacious and cost-effective. Most successful models have leveraged nurses, community health workers, and other non-physicians [14,15,16,17]. Recent studies demonstrate that programs for control of HIV can be leveraged for the control of chronic diseases such as HTN [18,19], though quantitative data on care linkage, blood pressure control, and other operational outcomes are scarce [20,21,22]. Pilot projects to date have been small and local in scope, with mixed results in linking HTN patients to care and achieving blood pressure control [2,3,4,23,24,25,26]. These inconsistent findings may be due to incomplete program fidelity: projects designed to screen and treat patients for HTN and CVD are not consistently implemented as intended, precluding accurate assessment of their impact. A recent systematic review found the fidelity of such projects ranged from 16–52%, with high-fidelity programs yielding more positive results [15]–demonstrating that fidelity is prerequisite to program efficacy.

The Sustainable East Africa Research in Community Health (SEARCH) study (NCT01864603) is a large cluster-randomized trial evaluating the impact of a multi-disease test-and-treat strategy on HIV incidence in rural Uganda and Kenya [27]. Adults attending community health campaigns (CHC) are offered HTN screening and follow-up care from nurses and supervising physicians as part of the SEARCH multi-disease approach [27,28,29,30,31]. Previous work has demonstrated SEARCH’s moderate success in HTN screening and treatment at select sites [29,30,31,32,33]. However, the fidelity of the SEARCH study has not yet been evaluated, limiting the ability to strengthen its efficacy. To better understand the results of the SEARCH model of dual HIV/NCD care, we performed a stepwise evaluation of the fidelity and efficacy of its program delivery for HTN care across 32 communities encompassing 95,000 adults in two HIV treatment arms in Uganda and Kenya. We defined a sequence of steps for program delivery, and evaluated SEARCH’s fidelity in delivering HTN care across this cascade.

## Methods

### Study setting and background

We defined the HTN cascade as a sequential process of blood pressure checks and interpretation, referral and linkage to follow-up care for apparent HTN, and subsequent medication and counseling, within 32 non-adjacent rural Ugandan and Kenyan communities, enrolled in the SEARCH Study [27], in three discrete clusters (ten around Mbarara, Western Uganda; ten around Mbale, Eastern Uganda; and 12 around Kisumu, Kenya). Each community had approximately 9,000–12,000 persons within approximately 20 villages (~50% adults age ≥18 years). As described previously [27,28,29,30,31,32,33], during the study’s initial year, each community first held a census to enumerate households (157,985 adults aged ≥18 across the three clusters). After the census, SEARCH hosted a community health campaign (CHC) offering universal adult screening, linkage to care, and treatment for HTN, HIV, and diabetes to which all enumerated households were invited, and 94,911 of the enumerated adults attended. Each adult CHC participant was queried regarding history of HTN; use of HTN medications; and HTN risk factors such as alcohol use and anxiety. Participants who screened positive for HTN, HIV, or diabetes were referred to care at a government health center at the third or fourth tier of the Ugandan health system (HCIII or HCIV) [34], or the equivalent non-dispensary health centers in the Kenyan health system [35]; each CHC was sited to be within walking distance of a corresponding such health center. Briefly, Ugandan Health Centers IV are equivalent to district hospitals, offering two or more physicians, inpatient and outpatient wards, and laboratories, whereas Health Centers III have one supervising physician and an outpatient ward with a laboratory; in Kenya, non-dispensary centers are analogous to Ugandan Health Centers III. There, patients received further evaluation, counseling, and medication from clinical officers supervised by SEARCH physicians.

### Screening for hypertension at community health campaigns (CHCs)

Following initial education on the campaign, and registration against community census enumerations performed by SEARCH, all adult CHC attendees were screened for HTN by two independent methods. First each attendee was to undergo a direct blood pressure check by a SEARCH nurse, as detailed below. Second a SEARCH staff member was to ask each attendee 1) if they were ever diagnosed with HTN and 2) if so, whether they have taken HTN medication in the last three months. SEARCH devised its own pair of criteria for defining an attendee as screening “positive” for HTN per that protocol: first, all persons who reported a history of HTN with recent medication use as above were deemed positive by history criteria, regardless of measured blood pressure. Second, all persons whose blood pressure, as checked by the SEARCH nurse on the day of the CHC, was consistently elevated, were deemed to screen “positive” for HTN regardless of their stated history. Specifically, SEARCH nurses used an electronic sphygmanometer (Omron 742) to check each attendee’s blood pressure on their upper arm as they sat in a chair. Adults whose measured pressure was greater than or equal to 140 mm Hg systolic or 90 mg Hg diastolic pressure on this test, underwent two additional blood pressure checks, at least one minute apart from each other and from the first test. If the lowest of the three screenings was greater than or equal to 140 mg Hg systolic or 90 mg Hg diastolic, SEARCH staff recorded a positive HTN screen. For purposes of integration and scale-up with the national health systems of Uganda and Kenya, we based these criteria on the Uganda Ministry of Health’s 2012 National Guidelines for the Management of Common Conditions [36], which defined hypertension as “a blood pressure of greater than 140/90 mm Hg on two or more separate occasions five minutes apart” on a single visit, as well as the Kenya Ministry of Health’s analogous 2009 guidelines, which use the same threshold but call for three “separate readings” [37]. This definition contrasts with ACC-AHA and other international guidelines that require two or more separate *visits* [38]; the Uganda Ministry of Health’s 2016 revision amended the guideline to state measurements must occur “on at least 2 or 3 occasions 1 week apart [39].”

Participants with a positive screen by either their measured blood pressure or self-reported use of HTN medication were to receive an appointment at a health center for follow-up, regardless of any current or prior HTN care they were separately receiving and regardless of their blood pressure level. All adults who met neither of these criteria were to be designated as screening negative for HTN, and to receive no referral to HTN care. SEARCH staff were also to measure the height, weight, blood glucose, and HIV status of all adult participants, among other factors.

### Managing hypertension at government health centers (HCs)

**Fig 1:**
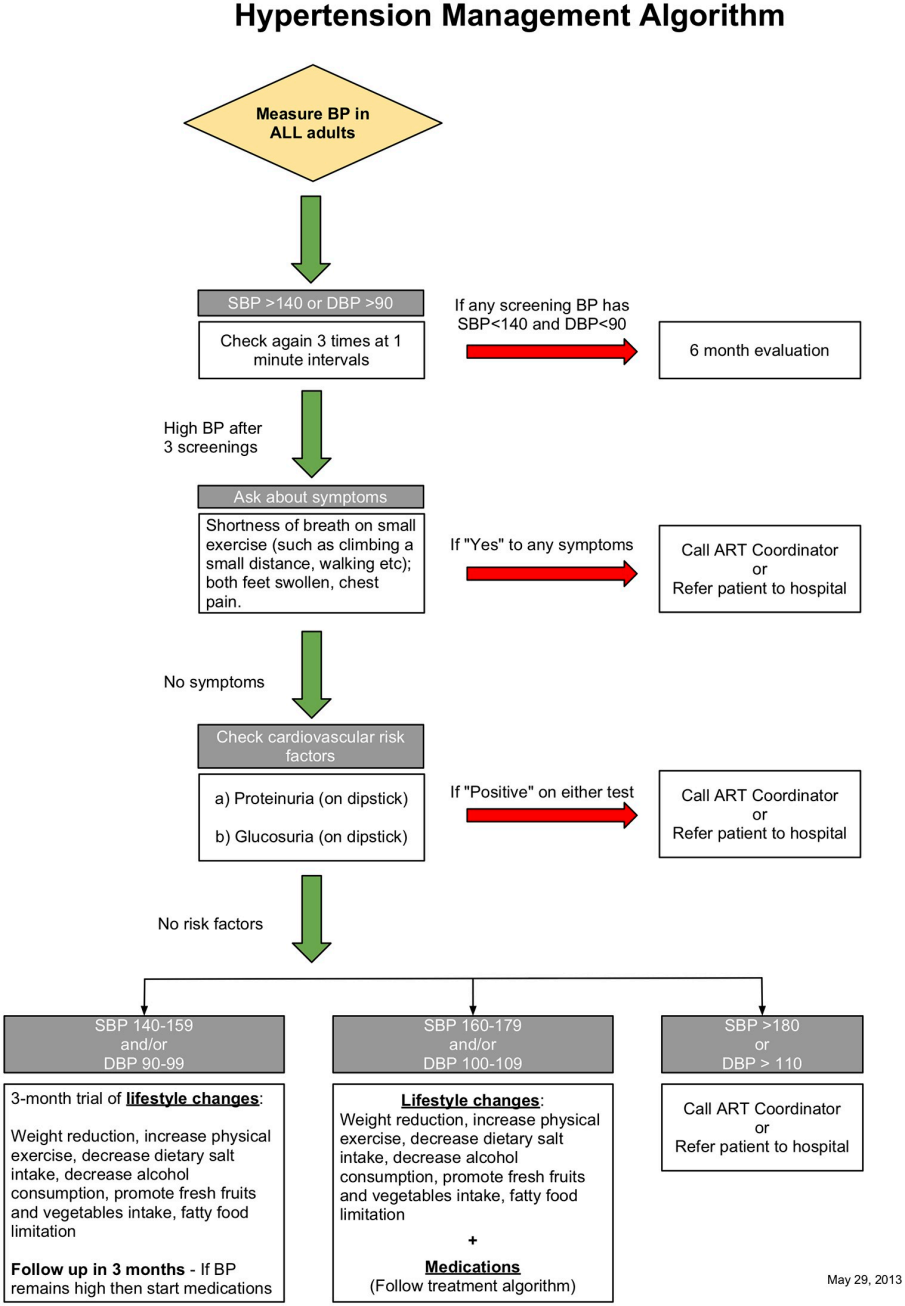
SEARCH health center hypertension clinical care algorithm.

Participants with a positive HTN screen were to receive an appointment at a health center (HC) corresponding to their CHC site, on a sub-clinic dedicated to patients with HTN and diabetes, called the chronic disease clinic. Participants who appeared at this clinic without an appointment also received care, if they were confirmed as SEARCH participants and reported a positive HTN screen at a CHC as above. As per guidelines at Ugandan HCIII, HCIV, and equivalent Kenyan non-dispensary clinics [36,37], general physicians and nurses provided front-line care as clinical officers at the NCD clinic—with telephone and in-person oversight from a SEARCH physician. All NCD clinic patients confirmed as SEARCH participants who reported a HTN+ screening result from a CHC were to receive a blood pressure check; screening for cardiovascular symptoms such as chest pain and shortness of breath; a urinalysis for protein and glucose; and lifestyle counseling and HTN medication, according to a SEARCH algorithm aligned with the Ugandan and Kenyan national guidelines (Fig 1), which call for a trial of lifestyle change followed by medication after 4–6 months of follow-up [37] and tracking every 3 months once pharmacological treatment is initiated [36]. The governments of Uganda and Kenya furnished HTN medications, with backup supply from SEARCH when unavailable, to promote program sustainability.

**Fig 2:**
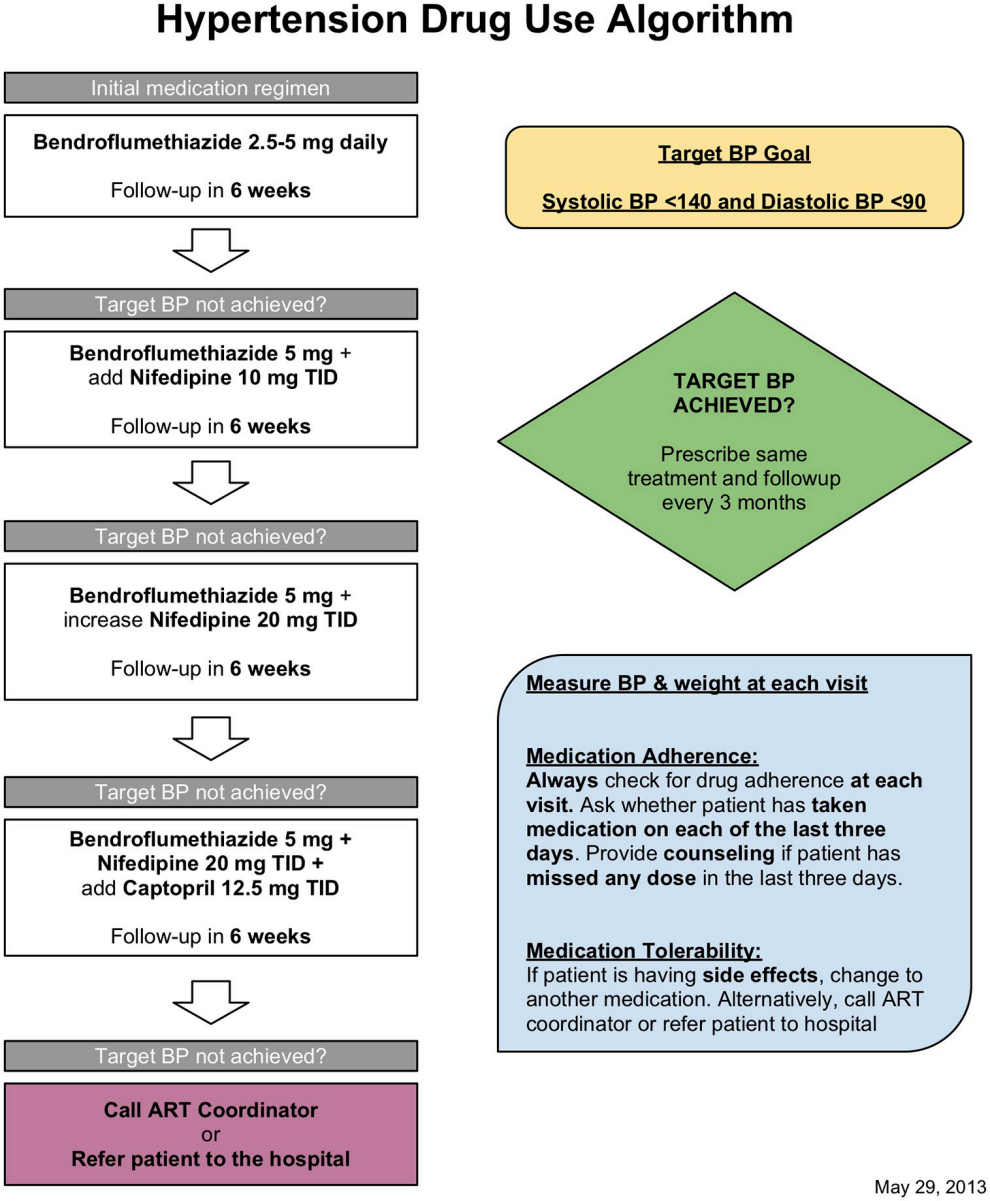
SEARCH health center hypertension medication treatment algorithm.

At their initial visit, participants with a blood pressure result under 140 mm Hg systolic and 90 mm Hg diastolic were to receive no intervention, only an appointment for a 6-month follow-up visit; those with a blood pressure 140-<160 mm Hg systolic or 90-<100 diastolic were to receive lifestyle counseling and a recheck at a later visit; and participants with blood pressure ≥160 mm Hg systolic or ≥100 diastolic were to receive HTN medication according to a stepwise algorithm (Fig 2). At follow-up visits, all participants with blood pressure that persisted at ≥140 mm Hg systolic or ≥90 mm Hg were to receive medication, even if not on medication previously. Providers were to augment the dose and type of medication until blood pressure control (systolic BP <140 mm Hg systolic and diastolic <90 mm Hg) was achieved; at that stage, participants were to receive refills of the same medication. In accordance with Ugandan and Kenyan national guidelines [36,37], treatment began with a thiazide diuretic, before transitioning to a calcium channel blocker or an angiotensin-converting-enzyme (ACE) inhibitor. Of note, neither of these guidelines advocated different blood pressure targets for older persons [36,37], and neither did SEARCH during the study period; however, SEARCH has since relaxed its blood pressure target of systolic BP<150 mm Hg and 90 mm Hg for patients over 60 [33] (Appendix) in alignment with international guidelines [38,39,40]. Similarly, blood pressure goals were the same for persons with and without diabetes, per Ugandan and Kenyan guidelines [36,37]. All participants taking medication were to be queried about medication adherence by 3-day recall in person at every clinic visit–this approach was designed to mirror medication adherence instruments validated for HIV care with which clinic staff were already familiar [41]. All participants, regardless of blood pressure control, were to receive follow-up appointments following each visit.

### Approach and analytic plan

We defined a stepwise cascade for evaluating the fidelity by which SEARCH conducted HTN-related activities based on the algorithm described above, measuring at each step the proportion of participants whose status was in fact measured as expected, and when possible, whether it was measured correctly: for example, whether SEARCH’s recorded interpretation of a participant’s HTN screen aligned with their actual blood pressure readings and stated history of HTN. We treated missing data, therefore, as itself a result rather than the absence of findings. However, in addition to evaluating SEARCH’s effectiveness in HTN care relative to care delivery intent (i.e., its fidelity to the algorithm above), we also measured its impact in achieving key results in HTN control (i.e., its effect on care outcomes), via a series of steps validated in HTN control studies globally [42,43]. The cascade sequence of fidelity steps is displayed in the Results section.

### CHC data analysis

Each SEARCH participant was invited to appear at a single CHC during the initial study year, but those who screened HTN+ were referred to HC care for multiple follow-up visits. Therefore, CHC analyses evaluated consistency of care per participant, whereas HC analyses evaluated consistency of care per visit. Because CHC data included both participants’ actual blood pressure and self-reported HTN status, as well as SEARCH staff interpretation of this status (i.e., whom SEARCH labeled as screening positive or negative for HTN), we calculated the sensitivity and specificity of SEARCH’s designation of HTN status, relative to the documented blood pressure and history values: in other words, SEARCH’s accuracy in interpreting the data it gathered.

Because all participants whom SEARCH deemed HTN-positive at each CHC were to receive an appointment to HC care, we calculated the proportion of participants whom SEARCH *labeled as HTN-positive*, as well as the proportion of participants whose blood pressure and history *was in fact confirmatory* for HTN, who received an appointment to HC care. Among participants who received an appointment, we calculated the proportion who appeared in a HC at least once (linked to care) between their CHC visit and May 2016. Because SEARCH operated separate, discrete research and clinical operations across the three regional village clusters (Eastern Uganda; Western Uganda; and Kenya), we stratified data across these three sites, as well as by body mass index (kg/m^2^) and diabetes status (12 or more mmol/L blood glucose on spot check).

### Health center data analysis

All SEARCH participants who presented to the HC were treated for HTN based on self-report of their CHC screen rather than CHC data, because the latter was unavailable to HC providers: at their first HC visit, participants were queried about whether they came to the clinic due to a positive screen for HTN, diabetes, or both; and their recall was presumed to be correct and duly recorded. We therefore calculated fidelity of HTN care for all persons reporting a positive HTN screen, regardless of prior CHC data. All SEARCH patient HC visits for HTN involved a blood pressure check; a query for HTN medication adherence; and a decision on medication treatment based on blood pressure and prior medication use (Fig 1). For these visits, we calculated the proportion in which blood pressure was checked and recorded and the proportion in which medication was prescribed appropriately based on SEARCH’s algorithm. We calculated each participant’s initial visit as appropriately-managed if a) blood pressure under 160 mm Hg systolic and under 100 mm Hg diastolic, and medication was not given; b) blood pressure was elevated above these thresholds and medication was given; or c) the participant reported prior medication use and medication was given. For all follow-up visits (the second or later for a given participant), we calculated as appropriately managed all visits in which a) blood pressure under 140 mm Hg systolic and under 90 mm Hg diastolic, and medication was not given; b) blood pressure was elevated above these thresholds and medication was given; or c) the participant reported prior medication use and medication was given. For all visits in which the participant reported medication use, we calculated whether medication adherence on all of the last three days was recorded (Fig 2). Because SEARCH did not record the results of HC patient symptom and urinalysis screens, we could not calculate how frequently clinicians checked these factors.

SEARCH required that all HTN participants at NCD clinics receive a follow-up within 6 weeks (Fig 2). We therefore calculated the proportion of visits in which participants received a follow-up appointment; and the percentage of follow-up appointments scheduled for under 42 days. Lastly, we calculated the proportion of participants who returned for at least one follow-up visit after their initial visit, and the proportion of participants who after two or more visits achieved a normal blood pressure at their most recent visit. As per above, we analyzed only visits in which the participant reported a positive CHC screen for HTN. A separate analysis of HC care among participants with a confirmed positive HTN screen at CHC is reported in the appendix. We presumed that all clinical data not recorded for a given visit (for example, blood pressure results) were not in fact collected. As with CHC data, we stratified data by SEARCH region, and by patient BMI and diabetes status. We used STATA 13.1 for analysis [44].

### Ethics

Heads of household provided written consent for all household members to participate in the SEARCH trial. We obtained verbal consent from all participants to participate in the CHC intervention. The Makerere University School of Medicine Research and Ethics committee; the Uganda National Council on Science and Technology; the Kenya Medical Research Institute Ethical Review Committee; and the University of California, San Francisco Committee on Human Research each approved the study.

### Role of the funding source

The study sponsors had no role in the study design, data collection, data analyses, data interpretation, or publication decisions associated with this study.

## Results

**Fig 3:**
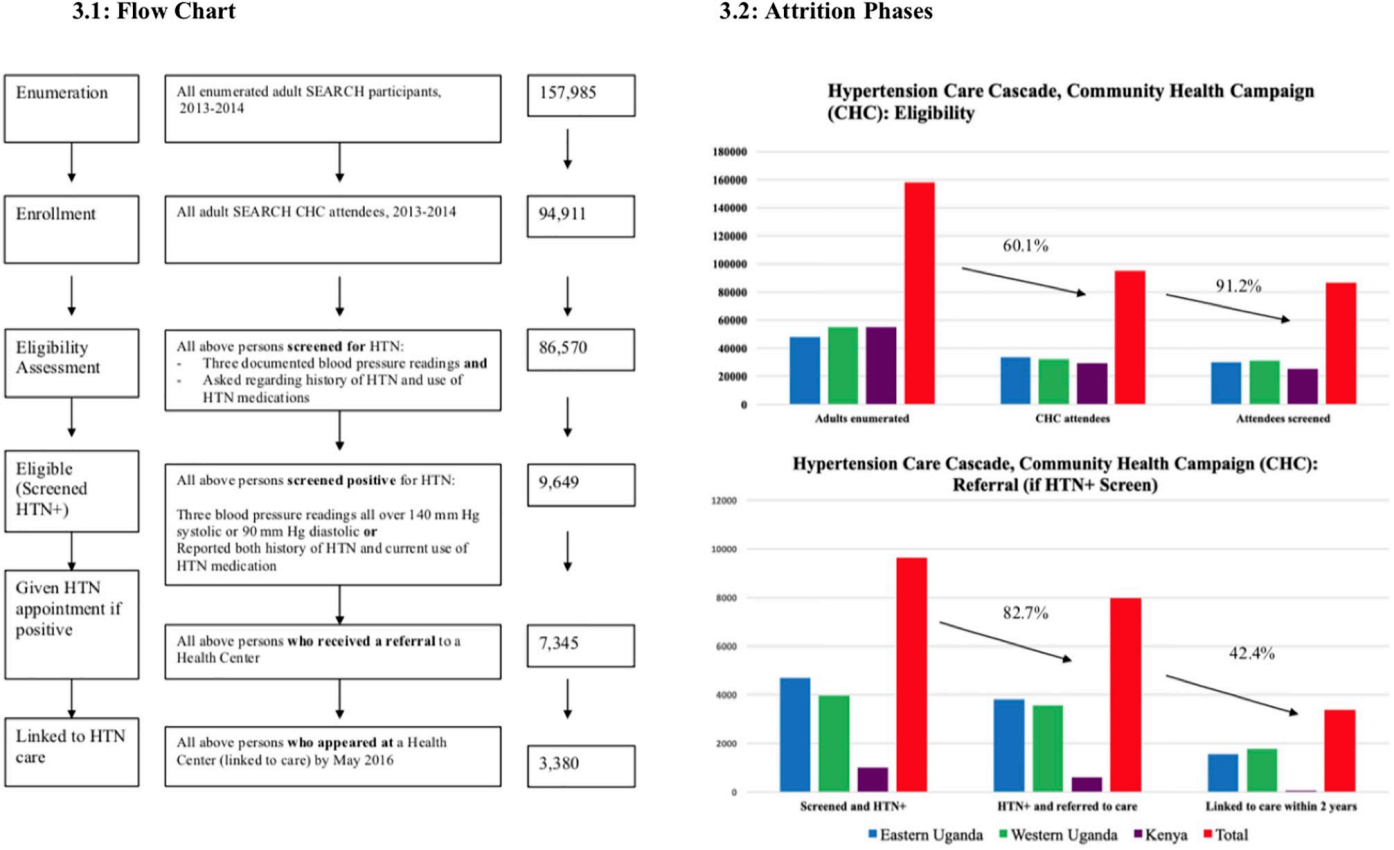
Consort diagram, fidelity, and outcome measures for CHC hypertension cascade. Flow chart depicts stepwise care cascade; bar graphs depict size of cumulative attrition.

**Fig 4:**
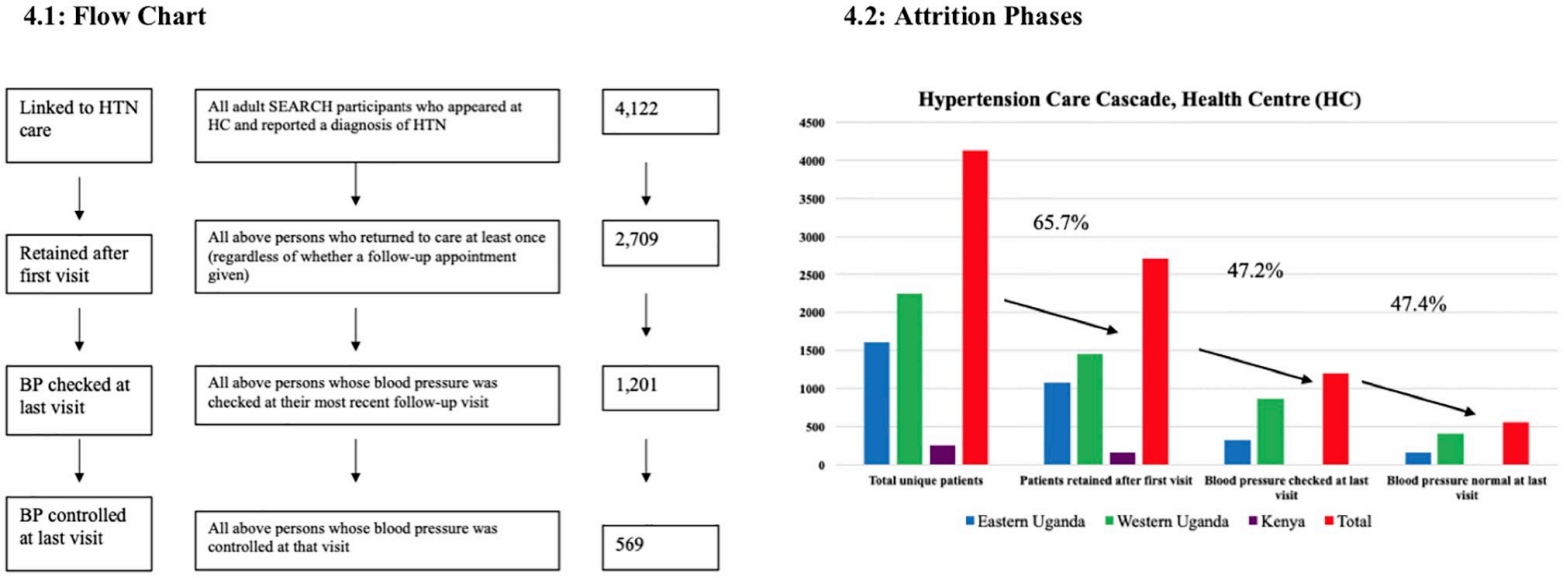
Consort diagram, fidelity, and outcome measures for health clinic hypertension cascade. Flow chart depicts stepwise care cascade; bar graphs depict size of cumulative attrition.

Participant demographics are displayed in Table 1, and a summary of the treatment cascade, and its stepwise attrition, in Figs 3 and 4. SEARCH enumerated a total of 157,985 adults across the three sites, of which 94,911 (60.1%) came to CHCs: 33,396 persons in Eastern Uganda; 32,250 in Western Uganda; and 29,265 in Kenya. Approximately 60% of participants in all three sites were female, with median age between 30 and 44. Most had a primary school education or less, including nearly 90% of participants in Kenya and 80% in Eastern and Western Uganda. Roughly 16% of persons across the regions were overweight or obese, and 15% underweight, with higher mean BMI in Eastern Uganda and lower in Western Uganda; blood glucose values consistent with diabetes were universally rare (0.5%). Among participants who received all blood pressure and history checks sufficient to screen them for HTN (i.e., as detailed above, between one and three direct measurements of blood pressure *and* queries regarding HTN history and HTN medication use), 16% screened positive in Eastern Uganda, 13% in Western Uganda, and 4% in Kenya. SEARCH recorded 14% of all participants as positive for HTN in Eastern Uganda and 12% in Western Uganda. In Kenya, SEARCH did not record its assessment of HTN or diabetes status, contrary to its intentions, due to limited staff and logistical capacity directed chiefly at the HIV/AIDS care cascade.

**Table 1:** CHC participant demographics, by subregion*.

Eastern Uganda N = 33,396 persons (of 47,911 total)		Western Uganda N = 32,250 persons (of 55,177 total)	Kenya N = 29,265 persons (of 54,897 total)	Total N = 94,911 persons (of 157,985 total)
**Gender**				
Male	14,083 (42.2%)	13,099 (40.6%)	10,566 (36.1%)	37,748 (39.8%)
Female	19,313 (57.8%)	19,151 (59.4%)	18,699 (63.9%)	57,163 (602%)
**Age**				
18–29	13,414 (40.2%)	11,999 (37.2%)	11,329 (38.7%)	36,742 (38.7%)
30–44	10,164 (30.4%)	10,426 (32.3%)	8,544 (29.2%)	29,134 (30.7%)
45–59	5,758 (17.2%)	5,856 (18.2%)	5,047 (17.2%)	16,661 (17.6%)
≥60	4,060 (12.2%)	3,969 (12.3%)	4,345 (14.8%)	12,374 (13.0%)
**Education Status**^1^				
No education	5,942 (17.4%)	7,114 (22.1%)	2,386 (8.2%)	15,442 (16.3%)
Education at primary level or below	19,639 (58.9%)	18,353 (56.9%)	23,768 (81.5%)	61,760 (65.2%)
Any secondary education or beyond	7,755 (23.3%)	6,767 (21.0%)	3,014 (10.3%)	17,536 (18.5%)
**Body Mass Index**^2^				
Underweight	4,546 (14.5%)	5,705 (18.0%)	3,764 (13.3%)	14,015 (15.3%)
Normal Weight	20,689 (65.9%)	21,346 (67.3%)	20,557 (72.4%)	62,592 (68.39%)
Overweight	4,940 (15.7%)	3,722 (11.7%)	3,235 (11.4%)	11,897 (13.0%)
Obese	1,235 (3.9%)	946 (3.0%)	838 (3.0%)	3,019 (3.3%)
**Diabetes**^3^				
No	31,168 (99.5%)	30,534 (99.5%)	n/a	61,702 (99.5%)
Yes	152 (0.5%)	157 (0.5%)	n/a	309 (0.5%)
**Confirmed HTN screen, by blood pressure or history**^4^	4,690 (15.5%)	3,955 (12.7%)	1,004 (4.0%)	9,649 (11.1%)

^1^. N = 33,336 E. Uganda; 32,234 W. Uganda; 29,168 Kenya.

^2^. Body mass index = weight in kilograms divided by square of height in meters. <18.5 = underweight; 18.5-<25 = normal weight; 25-<30 = overweight; </ = 30 = obese. N = 31,410 E. Uganda; 31,719 W. Uganda; 28,394 Kenya.

^3^. Diabetes defined by one-time glucose check 12 mmol/L or greater at CHC. N = 31,320 in E. Uganda and 30,361 in W. Uganda. Glucose check not performed in Kenya.

^4^. Gold standard = self-reported history of HTN with current medication use or three consecutive blood pressure values with each either SBP> = 140 mm Hg or DBP> = 90 mm Hg. Calculated relative to SEARCH listing of HTN+ screen. N = 30,243 E. Uganda; 31,069 W. Uganda; 25,258 Kenya because not all patients fully screened for HTN.

*Kruskal-Wallis equality-of-populations test performed on each demographic factor to compare sub-regions. Probability P of equal proportions across subregions = <0.05 for diabetes, and 0.0001 for all other factors.

Across the three CHC sites, 91% of all participants were fully screened for HTN (Fig 3), of which 9,649 (11.1%) screened positive, 7,978 (82.7%) received linkage to care, and 3,380 of these persons (42.4%) linked to care by May 2016. Of note, the rate of positive screening for HTN was significantly lower than the reported prevalence of HTN in Uganda and Kenya (25–30% or greater) [9,12,13], perhaps due to sample bias and/or limitations in the SEARCH measurement protocol as discussed below.

These results varied substantially across the three sites (Table 2). All but 18 of the 32,250 participants in Western Uganda were queried regarding prior history of HTN, and all but 78 of 33,396 participants in Eastern Uganda and 6 of 29,265 participants in Kenya. Proportions of blood pressure screening were lower: from 97% in Western Uganda to 86% in Kenya. 91% of attendees received all blood pressure checks and patient history checks sufficient to establish their HTN status (i.e. were fully screened) Relative to this confirmed result, the sensitivity of SEARCH’s recorded HTN assessment was 88% in Eastern Uganda and 94% in Western Uganda (and 91% overall), with specificity of nearly 100% at both sites. Among persons with a confirmed positive HTN screen, 83% received an appointment to attend an HC, including 60% in Kenya and 90% in Western Uganda. Within two years, 42% of persons participating in the initial-year CHCs had linked to care, including 50% in Western Uganda and 10% in Kenya.

**Table 2:** Community health campaign (CHC) implementation fidelity, by subregion*.

Eastern Uganda N = 33,396 persons		Western Uganda N = 32,250 persons	Kenya N = 29,265 persons	Total N = 94,908 persons
**History-Taking**				
Asked history of HTN	33,318 (99.8%)	32,212 (99.9%)	29,259 (100.0%)	94,789 (99.9%)
Asked if on medications, if HTN by history 1	2,077 (99.9%)	1,092 (100%)	1,202 (99.9%)	4,371 (100.0%)
**Examination:** Blood pressure checked 2	30,307 (90.8%)	31,104 (96.4%)	25,265 (86.3%)	86,676 (91.3%)
**Blood pressure and HTN history both checked**	30,243 (90.6%)	31,069 (96.3%)	25,258 (86.3%)	86,570 (91.2%)
**SEARCH-assessed HTN+, relative to confirmed HTN+:**				
Sensitivity	4131 true positive / 4690 true positive plus false negative: 88.1%	3,710 true positive / 3,955 true positive plus false negative: 93.8%	n/a	7,841 true positive / 8,645 true positive plus false negative: 90.7%
Specificity	25,477 true negative / 25,553 true negative plus false positive: 99.7%	27,085 true negative / 27,114 true negative plus false positive: 99.9%	n/a	52,562 true positive / 52,667 true positive plus false negative: 99.8%
**Linkage to Care:**				
Given appointment if assessed HTN+ by SEARCH 3	3,795 (89.3%)	3,550 (94.5%)	n/a	7,345 (91.7%)
Given appointment if confirmed HTN+ 4	3,819 (81.4%)	3,558 (90.0%)	601 (59.9%)	7,978 (82.7%)
Linked to HC care within two years if confirmed HTN and given appointment:	1,550 (40.6%)	1,770 (49.7%)	60 (10.0%)	3,380 (42.4%)

^1^. N = 2,080 E. Uganda; 1,092 W. Uganda; 1,203 Kenya.

^2^. One blood pressure check for all adult participants; two more if initial check elevated.

^3^. N = 4,250 E. Uganda; 3,758 W. Uganda

^4^. N = 4,690 E. Uganda; 3,955 W. Uganda; 1,004 Kenya.

* Kruskal-Wallis equality-of-populations test performed on each CHC factor to compare sub-regions. Probability P of equal proportions across subregions = <0.05 for blood pressure check and medication ask; and 0.0001 for all other factors.

At Health Centers, 66% of persons returned at least once after their initial visit (Fig 4), but only 44.3% had their blood pressure checked at their final follow-up visit, of which 52.6% achieved blood pressure control. This result included 67% in Eastern Uganda; 65% in Western Uganda; 64% in Kenya, and 66% overall (Table 3). Clinical care was largely consistent with SEARCH algorithms, although data on blood pressure, medications, and follow-up appointments were not recorded in Kenya. In Eastern and Western Uganda, 78% and 85% of HC visits involved a blood pressure check. SEARCH providers prescribed HTN medication as per SEARCH algorithms on 72% of visits in Eastern Uganda and 77% of visits in Western Uganda.

**Table 3:** Health center (HC) implementation fidelity, by subregion*.

Eastern Uganda N = 7,628 visits (1,615 persons)^1^		Western Uganda N = 10,639 visits (2,250 persons)	Kenya N = 892 visits (257 persons)	Total N = 19,159 visits (4,122 persons)
**Examination: at least 1 blood pressure checked at visit**	5,938 (77.8%)	9,052 (85.1%)	n/a^2^ 8	14,990 (82.1%)
**Assessment: Given medications appropriate based on examination**	5,520 (72.4%)	8,210 (77.2%)	n/a	13,730 (75.2%)
**Retention in Care:**				
Follow-up scheduled^3^	5,980 (78.4%)	8,876 (83.4%)	n/a	14,856 (81.3%)
Follow-up date in 6 weeks or fewer (if scheduled)	2,650 (44.3%)	4,205 (47.4%)	n/a	6,855 (46.1%)
Had follow-up visit at least once over two years (year 1)^4^	1,085 (67.2%)	1,460 (64.9%)	164 (63.8%)	2,709 (65.6%)
**Follow-up Care: If currently taking medication, asked about adherence**^5^	5,836 (93.9%)	7,049 (92.8%)	630 (70.6%)	13,515 (91.9%)
Blood pressure checked at most recent follow-up visit^6^	335 (30.9%)	866 (59.3%)	n/a	1,201 (47.2%)
**Blood pressure controlled at most recent follow-up visit**	164 (49.0%)	405 (46.8%)	n/a	569 (47.4%)

^1^. Refers to unique visits, not unique participants (most participants appeared multiple times). Refers only to persons treated within first year who self-reported a positive HTN screen at CHC.

^2^. Blood pressure values not recorded in Kenya.

^3^. All HC visits are to end in follow-up per SEARCH algorithm. Therefore N = 7,628 E. Uganda, 10,639 W. Uganda; 892 Kenya.

^4^. Among all persons who appeared at health center within 2 years of CHC visit. N = 1,615 E. Uganda; 2,250 W. Uganda; 257 Kenya.

^5^. Not all patients receive medication, per SEARCH treatment algorithm. N = 6,218 E. Uganda; 7,596 W. Uganda; 892 Kenya (all patients presumed taking medication).

^6^. Many patients did not receive a blood pressure check during their final HC visit and hence were not included

* Kruskal-Wallis equality-of-populations test performed on each HC factor to compare sub-regions. Probability P of equal proportions across subregions = >0.05 for two-year follow-up; >0.05 for blood pressure normal at last visit; and 0.0001 for all other factors.

Among persons who reported current use of antihypertensive medication, 93–94% were asked about adherence over the prior three days in Eastern and Western Uganda and 71% in Kenya. 78% of visits ended with the patient receiving a follow-up appointment in Eastern Uganda and 83% in Western Uganda, but only approximately half of these appointments were scheduled in under 6 weeks. Among persons who came to the health center at least twice and had a blood pressure checked on their final visit, just under half were controlled in Eastern and Western Uganda (49% and 47%, respectively). Notably, this result constitutes only approximately 22% of persons retained in care, because blood pressure was checked in only 44% of patients’ most recent follow-up visits (Fig 3).

Results also varied as a function of body mass index (BMI) and diabetes status, both at CHCs and HCs (Table 4). Persons missing BMI and diabetes status were significantly less likely to undergo blood pressure screening (5.4% and 86.8%, respectively) than the population overall; persons with overweight, obesity, and diabetes were more likely to screen positive for HTN, but not significantly more likely to be referred to care. Notably, persons with missing BMI were far less likely to link to CHC care (27.8%), while persons with missing diabetes status were far more likely (76.9%). At HCs, persons with overweight or obesity were more likely to remain in care, but less likely to have their blood pressure checked or controlled (Table 5). Persons with diabetes were more likely to remain in care and receive blood pressure checks, but less likely to achieve control. Persons missing BMI or diabetes data were less likely to remain in care or undergo blood pressure checks.

**Table 4:** Community health campaign (CHC) implementation fidelity, by BMI and diabetes status*.

BP checked		HTN+	Referred	Linked
**Total (94,911)**	86,570 (91.2%)	9,649 (11.1%)	7,978 (82.7%)	3,380 (42.4%)
**Underweight (14,015)**	13,110 (93.5%)	1,410 (10.8%)	1,207 (85.6%)	504 (41.8%)
**Normal Weight (62,592)**	59,317 (94.8%)	5,580 (9.4%)	4,636 (83.1%)	1,905 (41.1%)
**Overweight (11,897)**	11,175 (93.9%)	1,884 (16.9%)	1,512 (80.3%)	692 (45.8%)
**Obese (3,019)**	2,786 (92.3%)	733 (26.3%)	587 (80.1%)	269 (45.8%)
**BMI missing (3,388)**	182 (5.4%)	42 (23.1%)	36 (85.7%)	10 (27.8%)
**Diabetes (309)**	295 (95.5%)	111 (37.6%)	103 (92.8%)	71 (68.9%)
**No diabetes (61,709)**	57,730 (93.6%)	6,528 (11.3%)	5,364 (82.2%)	1,378 (25.7%)
**Glucose missing (32,893)**	28,545 (86.8%)	3,010 (10.5%)	2,511 (83.4%)	1,931 (76.9%)

*Kruskal-Wallis equality-of-populations test performed on each CHC factor to compare BMI and diabetes subgroups. Probability P of equal proportions across subregions = 0.067 for referral as a function of BMI; 0.17 for linkage as a function of BMI; 0.11 for referral as a function of diabetes status; and 0.0001 for all other factors.

**Table 5:** Community health campaign (CHC) implementation fidelity, by BMI and diabetes status*.

Retained		BP Checked	BP Controlled
	2,709 (65.7%)	1,201 (44.3%)	570 (47.5%)
Underweight (634)	386 (60.9%)	197 (51.0%)	86 (43.7%)
Normal Weight (2,242)	1,445 (64.5%)	654 (45.3%)	324 (49.5%)
Overweight (777)	551 (70.9%)	223 (40.5%)	104 (46.6%)
Obese (311)	227 (73.0%)	94 (41.4%)	38 (40.4%)
BMI missing (158)	100 (63.3%)	35 (35.0%)	18 (51.4%)
Diabetes (69)	61 (88.4%)	26 (100%)*	9 (34.6%)
No diabetes (1,395)	997 (71.5%)	179 (98.4%)	88 (49.2%)
Glucose missing (2,658)	1,651 (62.1%)	998 (39.9%)	473 (47.4%)

* Total population 4,122 persons. Kruskal-Wallis equality-of-populations test performed on each HC factor to compare BMI and diabetes subgroups. Probability P of equal proportions across subregions = 0.019 for retention as a function of BMI; 0.022 for BP check as a function of BMI; >0.05 for BP control as a function of BMI and as a function of diabetes status; and 0.0001 for all other factors.

The uncertainty associated with results across the treatment cascade increased cumulatively. Table 6 calculates the extent of this effect, tracking all persons who screened positive for HTN at CHCs through their linkage to HC care, blood pressure screening, and control outcome (see further details on this population in the Appendix table). Although 543 persons who screened positive for HTN at SEARCH CHCs, out of 9,469 total, enrolled and retained in HC care and achieved blood pressure control, the 95% CI for this result ranges between 452 and 648, a range of approximately 17–19%.

**Table 6:** Uncertainty of CHC and HC cascade estimates, by step and overall.

BP Checked		HTN+	Referred	Linked	Retained	BP Checked	BP Controlled
**Total (94,911)**	86,570 (91.2%)	9,649 (11.1%)	7,978 (82.7%)	3,380 (42.4%)	2,233 (66.1%)	1,172 (52.5%)	543 (46.3%)
**95% uncertainty CI, per step**	91.0%–91.4%	10.9%–11.4%	81.9%–83.4%	41.3%–43.5%	64.5%–67.7%	50.4%–54.6%	43.5%–49.2%
**Cumulative range of uncertainty**	86,399–86,741 (91.0%-91.4%)	9,449–9,850 (10.9%–11.4%)	7,741–8,218 (80.2%–85.2%)	3,196–3,571 (40.1%–44.8%)	2,060–2,416 (61.0%–71.5%)	1,039–1,318 (46.5%–59.0%)	452–648 (38.5%–55.3%)

## Discussion

Our work demonstrates that it is possible to leverage HIV test-and-treat interventions for the control of HTN across low-resource rural settings in sub-Saharan Africa, but it also suggests opportunities to improve the intervention fidelity—and care outcomes—of NCD interventions that leverage HIV care delivery. Approximately 90% of the 95,000 adults who presented to SEARCH’s community health campaigns received not only an initial blood pressure check, but two more if the initial check was elevated–showing that nurses can perform and act on this test reliably. Because almost 100% of those persons were also queried regarding prior HTN history, their assessed HTN status was confirmable by globally-established standards [38,39,43,45]. SEARCH staff largely interpreted these data correctly, assessing approximately 90% of persons with confirmed HTN as positive. Because the majority of those persons received a linkage appointment, SEARCH correctly referred a total of 7,978 people across the three sites: 82.7% of the proportion with confirmed HTN (Table 2). To our knowledge, although small studies have demonstrated the feasibility of using non-physician workers for HTN control in low- and middle-income countries [14,14,16,17], no population-wide HTN screening initiative on SEARCH’s scale has previously been evaluated for care fidelity or outcomes, although these initiatives have become more common in recent years [14,15,16,17,46,47,48].

Moreover, in Eastern and Western Uganda, SEARCH demonstrates that supervised non-physician providers can provide consistent clinical care for HTN according to established algorithms, and retain patients over time, achieving treatment rates comparable to high-income countries [49]. Patients SEARCH succeeded in linking and retaining in care received diagnosis and treatment at far higher rates than Uganda overall, where approximately 7–28% are aware of their diagnosis, 85% of diagnosed persons untreated, and 50% of treated persons were uncontrolled [9,10,11,12].

Despite these results, SEARCH’s HTN treatment outcomes were hampered by cumulative attrition across multiple stages of the HTN care cascade, including linkage to care, treatment initiation, and blood pressure control; and its blood pressure prevalence data is not consistent with nationwide surveys in Uganda and Kenya. SEARCH fully screened 86,676 persons for HTN across the three sites (91.3%); but only 9,649 (11.1%) screened positive, including only 1,004 (4%) in Kenya–substantially lower than population-level estimates of 25% in adults in this region [9,12,13], suggesting possible gaps in measurement protocols. Among the 9,649 identified as positive, 1,671 were lost to follow-up. And among the remaining 7,978, only 3,380 linked to HC care within two years. Among individuals in HC care, under 80% received documented appropriate treatment, and only roughly half received timely follow-up appointments (Table 3). Nevertheless, assuming an overall prevalence of HTN of 11.1% among the 94,908 persons screened, 35.0% of all HTN+ persons at SEARCH CHC campaigns linked to HC care: substantial relative to a setting where fewer than 4% of persons with HTN receive a diagnosis and care [7], but with ample room for improvement. The evaluation of HTN care in Kenya was limited because many elements of the HTN care cascade—such as the CHC assessment of HTN status; and the HC data on blood pressure levels, HTN treatment, and follow-up–were unrecorded. We record HTN data in Kenya, nonetheless, because our goal was to capture SEARCH’s efficacy in recording HTN data as an outcome, itself–in addition to documenting its fidelity and efficacy in acting on such data once recorded. Per report of SEARCH clinical officers, HTN medication stock-outs were more common in Kenya than Uganda sites, potentially hindering both treatment and retention rates.

Our study has several limitations. We used data on HTN care performed (such as blood pressure results or reported disease history) as a proxy for the care itself. It is therefore possible that in some cases, these data were spuriously entered into SEARCH records—but the test or intervention itself overlooked or incorrectly performed. Conversely, the absence of certain data does not necessarily mean the appraisal did not occur: SEARCH still referred the majority of persons with HTN to HCs for follow-up care. This result especially impaired our interpretation of the incomplete Kenya data as above. SEARCH has since prioritized standardized protocols for collection of HTN data, such that future analyses may better identify disparities in care. The instruments and equipment used by SEARCH were sometimes imperfect: the blood pressure cuffs were calibrated but not independently validated, for example; the 3-day medication recall instrument may not reflect long-term adherence thoroughly; and patients at HCs may not have accurately remembered their HTN status when asked at the initial visit. Additionally, our treatment cascade presumes a linear, stepwise progression of participants from one phase of treatment to the next, when in fact some patients were lost from elements of the cascade but returned for others. For example, not all patients who presented to care at HC had prior appointments or HTN screens- but SEARCH HC providers treated all self-identified HTN patients regardless (Fig 1); for this reason, a stepwise progression of unique patients through the entire cascade is presented only in the Appendix and in Table 6. As a result, SEARCH likely treated more patients with true HTN than otherwise possible, but also some false-positives with normal blood pressure. Data on HC outcomes for the SEARCH patients who screened positive for HTN at a CHC and received an appointment (see final row, Table 2), summarized in the appendix, did not substantially differ from HC outcome data among persons with self-reported HTN (Table 4). Furthermore, we presumed that all persons who did not present to HC care went untreated–when many may have self-referred to other follow-up care. We captured only persons who presented to CHCs (60% of the total adult population) who may have differed systematically from those who did not (e.g., by having lower blood pressure, as reflected in our prevalence result). Additionally, we did not record cost data, and hence lack results on the intervention’s cost-effectiveness.

Lastly, the complexity (and ambiguity) of the SEARCH HTN care algorithm sometimes precluded a one-directional sequential analysis. For example, SEARCH trained HC providers not to give a HTN patient medication at their initial visit unless blood pressure exceeded 160 mm Hg systolic or 100 mm Hg diastolic (Fig 2). Patients with blood pressure 140–160 mm systolic Hg or 90–100 mm Hg diastolic should receive medication only at follow-up visits. However, it is unclear whether a patient with an initially normal blood pressure, followed by a blood pressure 140–160 mm systolic Hg or 90–100 mm Hg at a subsequent visit, should receive treatment at that visit (our analysis presumes yes), or whether a patient with self-reported HTN with multiple normal blood pressure visits should be discharged from care as a false-negative case (our analysis presumes no). Similarly, we did not examine sequential escalation of medications per SEARCH’s algorithm (Fig 2) but simply whether any SEARCH HTN medication was prescribed based on the patient’s blood pressure and prior medications. The complexity of the sequential SEARCH algorithm also magnifies uncertainty in reported results at each stage of intervention (Table 6), although even after seven such steps, the 95% confidence interval lies within 20% of the reported value, due to the study’s large underlying sample size.

Our findings suggest several means to better improve the fidelity, and outcomes, of HTN care through studies like SEARCH. For example, direct observation of providers at CHCs and HCs, and more detailed instruments to record their clinical findings, would permit more accurate evaluation of care delivery. Interviews with providers and patients could identify underlying barriers to care linkage and treatment, such as inconsistent counseling messages or delays in scheduling follow-up. In the past two years, SEARCH launched several participant-oriented reforms including faster appointments; new education materials, and appointment reminder cards; evaluation of these interventions is ongoing.

More broadly, however, this analysis defines a framework to evaluate the fidelity of screening and treatment for HTN, the leading preventable risk factor for death and disability—in a resource-limited setting requiring leveraging non-physician health workers and existing health delivery programs designed for HIV. A recent systematic review [16], which incorporated prior findings from SEARCH, demonstrated the resources and strategies most conducive to integrated care for HIV/AIDS and chronic disease—for example, patient-centered delivery models and a clear implementation strategy–and called for standardized care checklists and service coordinators to achieve it. However, other recent reviews of low-resource CVD care–including those leveraging HIV programs—report unclear and incompletely implemented treatment protocols, which impairs measuring and achieving consistent outcomes [16,16,20,21].

Our work developed a sequential “cascade” for the implementation and evaluation HTN care, derived from cascade models now aiding universal delivery of HIV treatment in multiple settings [50,51]. We have demonstrated this cascade is applicable across a large, multi-site HTN initiative that provides care from asymptomatic screening to outpatient continuous treatment, and identified elements, such as revised documentation, to improve its monitoring and evaluation. Moreover, we have demonstrated that–with some exceptions–the SEARCH integrated HIV-NCD model can be consistently delivered at scale in 32 diverse communities across two country settings. Additionally, we have found ample opportunities to improve both delivery fidelity and consequent clinical outcomes- using a model leveraging elements such as patient-centered delivery (local, free mass CHC screening) and health system coordination (physician oversight of health workers) shown to work in prior literature on HIV-NCD integration. As the burden of HTN continues to rise worldwide, this cascade model for HTN care fidelity, and the care structure that it guides, could serve as a model to aid HTN initiatives to achieve greater care delivery targets, perhaps in line with the “90-90-90” [50,51,52] goal for HIV case detection, treatment, and virologic suppression that now drives most cascade models for HIV care.

## Supporting information

S1 Appendix Health center data for persons screened HTN+ at CHCs and referred to health centers, by subregion. (DOCX)

S2 Appendix Revised SEARCH health center hypertension medication regimen algorithm, June 2015. (DOCX)

S1 Dataset (XLSX)
