# Supplementary material for: Hypertension testing and treatment in Uganda and Kenya through the SEARCH study: An implementation fidelity and outcome evaluation
Source: PLoS One. 2020 Jan 15;15(1):e0222801. doi: 10.1371/journal.pone.0222801 (PMC6961918; doi:10.1371/journal.pone.0222801)
Supplement: S1 Appendix — (DOCX) [file pone.0222801.s001.docx]

**Appendix 2: Revised SEARCH Health Center Hypertension Medication Regimen Algorithm^33^**
