# Supplementary material for: Hypertension testing and treatment in Uganda and Kenya through the SEARCH study: An implementation fidelity and outcome evaluation
Source: PLoS One. 2020 Jan 15;15(1):e0222801. doi: 10.1371/journal.pone.0222801 (PMC6961918; doi:10.1371/journal.pone.0222801)
Supplement: S2 Appendix — (DOCX) [file pone.0222801.s002.docx]

**Appendix 1: HC data for persons HTN+ at CHCs and referred to HCs, by Subregion:**

| **Eastern Uganda**  **N = 7,159 visits (1,550 persons) ^1^** | | **Western Uganda**  **N = 8,823 visits (1,770 persons)** | **Kenya**  **N = 251 visits (60 persons)** | **Total**  **N = 16,233 visits (3,380 persons)** |
| --- | --- | --- | --- | --- |
|  |  |  |  |  |
| **Examination: at least 1 blood pressure checked at visit** | 5,943 (83.0%) | 7,674 (87.0%) | n/a | 13,617 (85.2%) |
|  |  |  |  |  |
| **Assessment: Given medications appropriate based on examination** | 5,384 (75.2%) | 6,812 (77.2%) | n/a | 12,196 (76.3%) |
|  |  |  |  |  |
| **Retention in Care:** |  |  |  |  |
| Follow-up scheduled^2^ | 5,991 (83.7%) | 7,533 (85.4%) | n/a | 13,524 (84.6%) |
| Follow-up date in 6 weeks or fewer (if scheduled) | 2,562 (42.8%) | 4,025 (53.4%) | n/a | 6,587 (48.7%) |
| Had follow-up visit at least once over two years (year 1)^3^ | 1,038 (67.0%) | 1,150 (65.0%) | 45 (75.0%) | 2,233 (66.1%) |
|  |  |  |  |  |
| Follow-up Care: If currently taking medication, asked about adherence ^4^ | 4,832 (94.9%) | 5,945 (94.0%) | 173 (68.9%) | 10,950 (93.9%) |
| Blood pressure checked at most recent follow-up visit ^5^ | 455 (43.8%) | 717 (62.3%) | n/a | 1,172 (53.6%) |
| Blood pressure controlled at most recent follow-up visit | 233 (51.2%) | 310 (43.2%) | n/a | 543 (46.2%) |

1. Refers to unique visits, not unique participants (most participants appeared multiple times). Refers only to persons treated within first year with confirmed HTN at CHC and referral to HC who appeared at HC.

2. All HC visits are to end in follow-up per SEARCH algorithm. Therefore N = 7,159 E. Uganda, 8,827 W. Uganda; 251 Kenya.

3. Among all persons who appeared at health center within 2 years of CHC visit. N = 1,550 E. Uganda; 1,770 W. Uganda; 60 Kenya.

4. Not all patients receive medication, per SEARCH treatment algorithm. N = 5,089 E. Uganda; 6,326 W. Uganda; 251 Kenya (all patients presumed taking medication).

5. Many patients did not receive a blood pressure check during their final HC visit and hence were not included.

* Kruskal-Wallis equality-of-populations test performed on each HC factor to compare sub-regions. Probability P of equal proportions across subregions = 0.0293 for appropriate medication use; >0.05 for receiving a follow-up appointment; >0.05 for two-year follow-up; 0.0213 for blood pressure normal at last visit; and 0.0001 for all other factors.
